# Academy of Medicine Notes

**Published:** 1893-06

**Authors:** 


### Academy of Medicine Notes.

FELLOWS of the Academy desiring application blanks for their friends may obtain them by applying to the Secretary.

PHYSICIANS in Buffalo to be "on time" must belong to the Academy of Medicine, otherwise they must be classed as "late."

AT ITS last meeting, the Council appointed Frederic W. Bartlett, M. D., Librarian, and Herbert U. Williams, M. D., Curator of the Museum.

THE Section on Obstetrics and Gynecology elected the following officers at its last meeting : Dr. M. A. Crockett, President ; Dr. Lillian C. Randall, Secretary.

THE names of all members of the old societies, now comprising the Academy, who shall not have paid their initiation fee by June 21st, will be dropped from the Secretary's rolls. They can then only be admitted to the Academy through the Council.

THE next meeting of the Surgical Section occurs May 30th. The subject under discussion will be on Hemorrhoids, by Dr. Edward

G. Meyer ; Treatment of Hemorrhoids, by Dr. Edward Clark. This will be the last meeting of this Section until September 5th.

---

THE Section on General Medicine elected the following officers at its May meeting : Dr. John H. Pryor, President ; Dr. Electa B. Whipple, Vice-President ; Dr. J. H. Dowd, Secretary and Treasurer. This Section will not meet in June, the next meeting being in September.

---

ANYBODY doubting the wisdom of the movement which resulted in the formation of the Academy, should go to the Academy rooms on a Tuesday night and observe the change which has come over the Buffalo medical meetings. Pleasant rooms, good attendance, spirited discussions, and a hearty welcome, are symptoms which were not discoverable in the old societies.

---

THE next stated meeting of the Academy will be held June 20th, under the auspices of the section on Obstetrics and Gynecology. The annual meeting and the election of officers will occur June 27th. The nominations made at the March meeting of the Academy were : For President, Thomas Lothrop, M. D.; Treasurer, Eugene A. Smith, M. D.; Trustee, Frederic W. Bartlett, M. D.

